# Statistics of the Medical Colleges of the U. S., for the Session of 1847-8

**Published:** 1849-12

**Authors:** 


*Statistics of the Medical Colleges of the U. S., for the Session of* 1847-8.

| | Number of Students. | Number of Graduates. |
|---|---|---|
| University of Pennsylvania, | 499 | 199 |
| Medical College of Harvard University, Boston, | 126 | 25 |
| College of Physicians and Surgeons, New York, | 176 | 39 |
| Medical Institute of Yale College, | | |
| Medical School of Maine (Brunswick), | 74 | 14 |
| Vermont Medical College (Woodstock), | 105 | |
| Medical College of Castleton, Vermont, | | 23 |
| Dartmouth Medical College (N. H.), | | |
| Berkshire Medical Institution, | | 26 |
| Med. Dep. Univ., City of New York, | 412 | 134 |
| Albany Medical College, | 101 | 22 |
| Geneva Medical College, | 63 | 18 |
| Med. Dep. University of Buffalo, | 93 | 19 |
| Jefferson Medical College, | 497 | 188 |
| Med. Dep. Pennsylvania College, | | 36 |
| Philadelphia College of Medicine, | 91 | 21 |
| University of Maryland, | 190 | 94 |
| Washington University of Baltimore, | | |
| Med. Dep. of Columbian College (Washington), | | |
| University of Virginia, | | |
| Med. Dep. of Hampden Sidney College (Richmond), | | |
| Med. College of State of South Carolina, | 141 | 45 |
| Med. College of Georgia (Augusta), | 133 | 36 |
| Med. Dep. of Univ. of Louisiana (New Orleans), | | |
| Memphis Medical College, | | |
| Med. Dep. of Transylvania College, | 120 | 46 |
| Med. Dep. University of Louisville, | 331 | 81 |
| Med. College of Ohio (Cincinnati), | 175 | 50 |
| Starling Medical College (Columbus), | 173 | 50 |
| Med. Dep. Western Reserve College (Cleveland), | 251 | |
| Indiana Medical College (Laporte), | | |
| Med. Dep. Illinois College, | | |
| Rush Medical College (Chicago), | 100 | 18 |
| Med. Dep. St. Louis University, | 102 | 22 |
| Med. Dep. University of Missouri (St. Louis), | 154 | 38 |
| Indiana Central Medical College | | |

—*American Journ. Med. Sciences.*

